# Exploring the causal relationship between immune factors and chondrosarcoma: a Mendelian randomization study

**DOI:** 10.1007/s12672-025-02654-5

**Published:** 2025-05-18

**Authors:** Taichuan Xu, Wentao Xiao, Wenjie Li, Xianfa Xu, Haiwen Zhang, Xian Zhang

**Affiliations:** 1 Nanjing University of Chinese Medicine, Nanjing, 210023 Jiangsu China; 2 Department of Spine, Wuxi Affiliated Hospital of Nanjing University of Chinese Medicine, Wuxi, 214072 Jiangsu China

**Keywords:** Chondrosarcoma, Immune trait, Inflammatory proteins, Mendelian randomization

## Abstract

**Background:**

Previous studies have investigated the potential role of immune factors in chondrosarcoma (CHS). However, the causal relationship is unknown.

**Methods:**

A two-sample Mendelian randomization (MR) was used to explore potential correlations between 731 immunocyte phenotypes, 91 inflammatory proteins, and CHS. The data were derived from published summary statistics of genome-wide association studies. Inverse-variance weighted was employed as the primary method. Furthermore, a range of analytical methods, including MR-Egger, weighted mode, and weighted median was used to enhance the robustness of the results. A two-step MR was used to assess the mediating effects of inflammatory proteins. Subsequently, sensitivity and MR Steiger directionality tests were performed.

**Results:**

MR analyses showed that 12 immunocyte phenotypes were positively correlated with CHS (*P* < 0.05, OR > 1), and 11 immunocyte phenotypes were negatively correlated with CHS (*P* < 0.05, OR < 1). Five inflammatory proteins were positively associated with CHS (*P* < 0.05, OR > 1). No heterogeneous or horizontal pleiotropy was found. The MR Steiger analysis found no statistically significant evidence of reverse causation. Mediation analysis did not identify any potential mediating effects.

**Conclusion:**

Our study underscores the pivotal role of immune factors in CHS and offers insights that can inform future research.

**Supplementary Information:**

The online version contains supplementary material available at 10.1007/s12672-025-02654-5.

## Introduction

Chondrosarcoma (CHS), a mesenchymal neoplasm characterized by malignant hyaline cartilage production, represents 20–25% of primary bone malignancies and ranks as the second most prevalent non-hematologic skeletal malignancy following osteosarcoma [1]. Patients predominantly manifest localized osseous pain or arthralgia characterized by insidious progression, and is frequently accompanied by an increase in pain at night [2]. The prevalence of CHS in the United States is estimated to be approximately 1 in 200,000 [3]. Its prevalence is increasing and it is regarded as the most prevalent primary bone malignancy in numerous countries [4, 5]. Surgical intervention remains the preferred treatment modality for patients with CHS. However, patients who are not candidates for surgical intervention due to unfavorable tumor location, large tumor size, or the presence of metastasis face significant clinical challenges [6]. In addition, this mesenchymal malignancy demonstrates marked chemoradiotherapy resistance due to its hypoxic microenvironment and intact DNA repair mechanisms. Patients with high-grade CHS often face the challenge of local recurrence and distant metastases after surgical resection while radical-wide resection may result in significant patient dysfunction [7]. Contemporary advances in cancer immunotherapeutics are redefining treatment paradigms across diverse malignancies. Emerging evidence between chondrosarcoma pathobiology and tumor-immune microenvironment remodeling underpins the therapeutic rationale for immunotherapy as a transformative strategy to overcome therapeutic resistance in CHS management [8].

Similar to other osteosarcomas, immune cells engage in extensive intercellular communication, facilitating tumor growth through the secretion of cytokines and the deposition of the extracellular matrix (ECM). Increased aggressiveness is a hallmark of the tumor microenvironment (TME) in CHS [9]. It has been demonstrated that CHS tumor cells are intimately intertwined with immune cells, and their TME is characterized by the presence of immune infiltrates, including macrophages, lymphocytes, and neutrophils [10]. Tumor-associated macrophages (TAM) are considered the predominant immune cell type within the CHS immune milieu and have been implicated in promoting tumor cell proliferation and chemoresistance [11]. Furthermore, accumulating evidence has consistently shown a well-established association between cytokine dysregulation and CHS pathogenesis. The elevated expression of colony-stimulating factor 1 receptor (CSF1R) in TAM within CHS binds to the corresponding ligand secreted by CHS cells, thereby driving the transformation of the immune microenvironment into a pro-tumorigenic phenotype [12]. Consequently, CSF1R inhibitors are emerging as promising therapeutic agents for CHS. Similarly, several clinical trials have investigated the potential efficacy of Programmed death-1 (PD-1) inhibitors in treating of CHS [13]. Nevertheless, it is important to note that not all observational reports have yielded consistent results. Li et al. revealed a scarcity of macrophages and monocytes in CHS tumor tissue, suggesting that immunotherapy targeting TAM may not be an appropriate strategy [14]. The molecular basis of CHS and its associated immune microenvironment remain poorly understood, with some studies reporting contradictory findings. Experimental studies depend on animal and patient-derived models, but the lack of representational near-patient preclinical models limits predictive drug screens [15]. This underscores the ongoing need for further research into the potential of immunotherapy for CHS [7].

Mendelian randomization (MR) employs genetic variation as an instrumental variable (IV) to ascertain causal relationships between exposure and outcome. The potential for confounding factors and reverse causality is minimized due to the random assignment of alleles from parent to offspring, with an individual’s genetic makeup being established at the time of fertilization [16]. In this study, we sought to elucidate the causal relationship between CHS and immune traits, as well as inflammatory proteins using two-sample MR analysis. Furthermore, we explored whether inflammatory proteins mediate the causal relationship between immune traits and CHS using a two-step MR analysis. We hope that this gene prediction method will provide insight into the pathogenesis and treatment of CHS.

## Materials and methods

### Study design

**Fig. 1:**
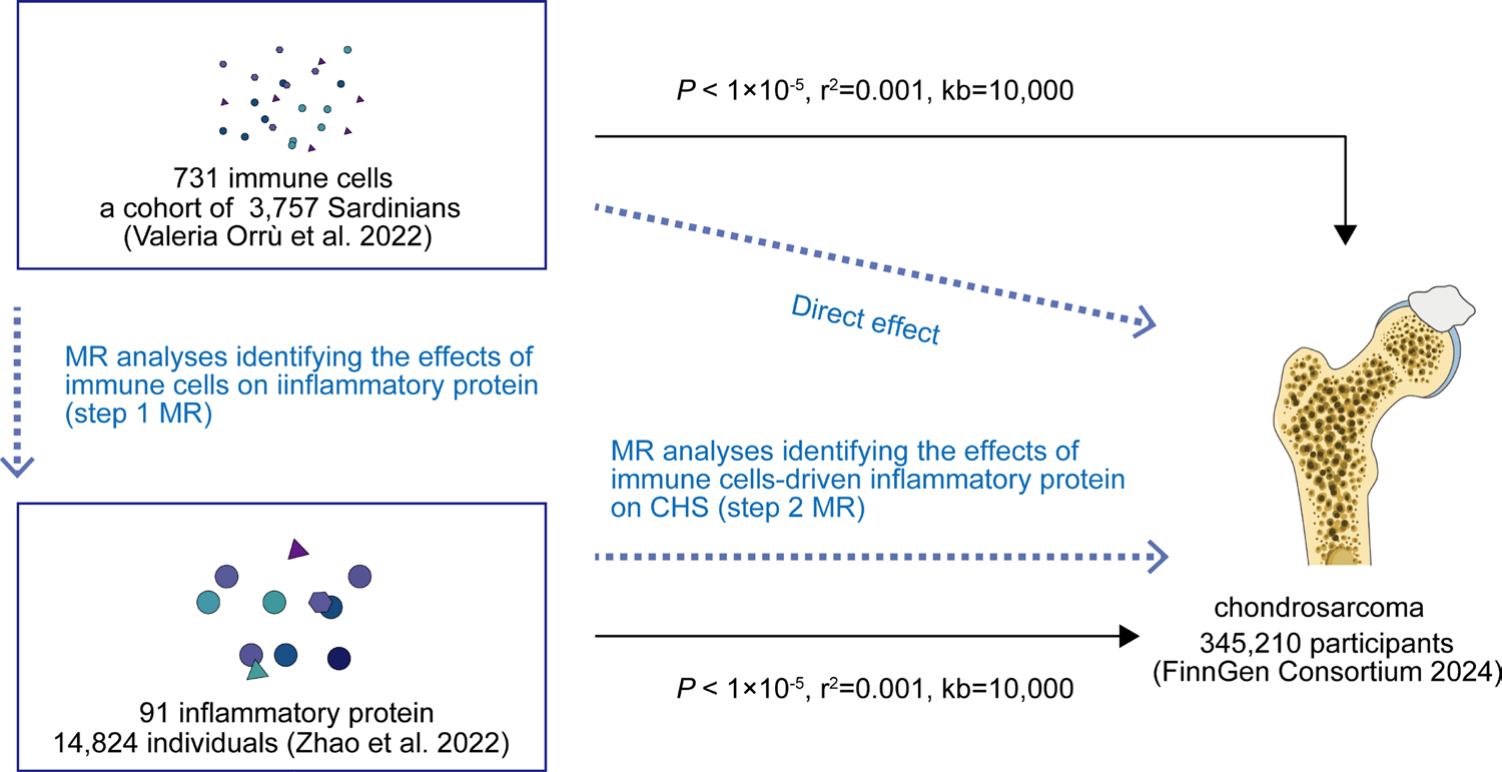
The workflow of this study

Firstly, we investigated the causal relationship between CHS and 731 immune traits, as well as 91 inflammatory proteins using two-sample MR. Due to insufficient IVs for inflammatory proteins, we were unable to conduct reverse MR. To determine the direction of potential causality, we conducted an MR Steiger test [17]. Subsequently, we employed mediation analyses to elucidate the mediating effects of inflammatory proteins on the causal impact of immune traits on CHS. Our study was conducted in accordance with the principles of relevance, independence, and exclusivity. The IV was strongly associated with exposure and was not associated with confounding factors. Furthermore, IVs affect outcomes exclusively through the exposure factors [16]. No further ethical approval was required, as the study utilized publicly available genome-wide association data. Our MR study followed the STROBE-MR guidelines [18]. The checklist is summarized in Supplementary Table 1. The comprehensive design of the study is illustrated in Fig. 1.

### Data source

We selected the largest and most up-to-date genome-wide association studies (GWASs) summary data available for immunocyte phenotypes, inflammatory proteins, and CHS, taking into account the need to avoid ethnic stratification and sample overlap. The GWAS catalog provides data on immunophenotypic characterization, which are accessible through the IEU Open GWAS database (https://gwas.mrcieu.ac.uk/), with GWAS IDs ranging from GCST90001391 to GCST90002121. Valeria Orrù and colleagues conducted whole-genome sequencing of approximately 22 million genetic variants in 3,757 individuals from Sardinia, generating GWAS statistics for immune traits across four trait types [382 median fluorescence intensities (MFI) for surface antigens; 192 relative cell (RC) counts; 118 absolute cell (AC) counts; and 32 morphometric parameters (MP)] and seven panels of immune phenotypes (Monocyte, B cell, Treg, TBNK, Maturation stages of T cell, cDC, Myeloid cell) [19]. Data on inflammatory proteins were obtained from a study employing the Olink-Targeted Inflammation Panel, which included 14,824 individuals across 11 European descent cohorts [20]. Full per-protein GWAS summary statistics are available for download at https://www.phpc.cam.ac.uk/ceu/proteins and the EBI GWAS Catalog (accession numbers GCST90274758 to GCST90274848). For CHS, GWAS data from the Finnish Genetic (FinnGen) Consortium, based on the C3_CHONDROSARCOMA_EXALLC dataset and published in 2024, was selected (https://www.finngen.fi/en/access_results). The dataset encompassed 345,210 participants of European ancestry, comprising 92 cases and 345,118 controls, and included a total of 20,092,308 single-nucleotide polymorphisms (SNPs) [21].

### Selection of instrumental variables

We screened the IVs by referring to previous studies. To filter the exposure data, a significance threshold of *P* < 1 × 10⁻^5^ was set for relevant SNPs [22–24]. To address potential confounding by linkage disequilibrium (LD), we implemented a clumping procedure (r^2^ threshold = 0.001) with a 10,000 kb genomic window based on standard GWAS quality control protocols. To minimize the potential for weak IV bias, we excluded IVs with *F*-statistics [$F={R}^{2}\left(N-K-1\right)/K\left(1-{R}^{2}\right)$] of less than 10. R^2^ denotes the proportion of exposures explained by each SNP, N represents the sample size for each exposure, and K is the number of SNPs included in the IV. Subsequently, the exposure dataset was merged with the resultant dataset to facilitate further analysis. Ambiguous SNPs with incongruent alleles and palindromic SNPs with ambiguous strands were corrected. SNPs that could not be harmonized were excluded [25]. We endeavored to determine the direction of causal inference through reverse MR. However, since we were unable to screen for the IVs utilized in the analysis, we determined the direction of causal inference using the MR Steiger test [17].

### Mendelian randomization analysis and sensitivity analysis

The random-effects inverse-variance weighted (IVW) method was utilized as the principal analytical framework, providing robust causal inference under MR assumptions when horizontal pleiotropy remains balanced [25]. The weighted median, weighted mode, and MR-Egger methods, which are robust to horizontal pleiotropy, were employed as complementary approaches to the IVW [26]. Our MR analysis was conducted as an exploratory study, and the results were considered nominally significant at *P* < 0.05 [27]. We explored the mediating role of immune factors in CHS through mediation analysis. We first identified significant causal associations between exposure and CHS, and subsequently estimated the mediating effects of inflammatory proteins using the coefficient product method. Genetic instruments of exposure were used to estimate their causal effects on potential mediators. Thereafter, the effects of the screened inflammatory proteins on CHS were evaluated [28]. The mediating effect was calculated by multiplying the effect of exposure on the mediator by the effect of the mediator on the outcome. The standard error of the mediating effect was estimated using the delta method. The proportional contribution of the mediating effect was then calculated by dividing the mediating effect by the total effect of the exposure on the outcome [29]. Statistical analyses were performed using R software (version 4.3.0), TwoSampleMR package (version 0.5.6), and RMediation package (version 1.2.2). The MR-Egger intercept test and MR-PRESSO test were used to employed potential horizontal pleiotropy. Cochran's Q test within the IVW framework was used to detect heterogeneity. Leave-one-out sensitivity analysis was conducted to determine whether the causal effects were driven by specific SNPs.

## Results

### The causal relationship between immune traits and CHS

**Fig. 2:**
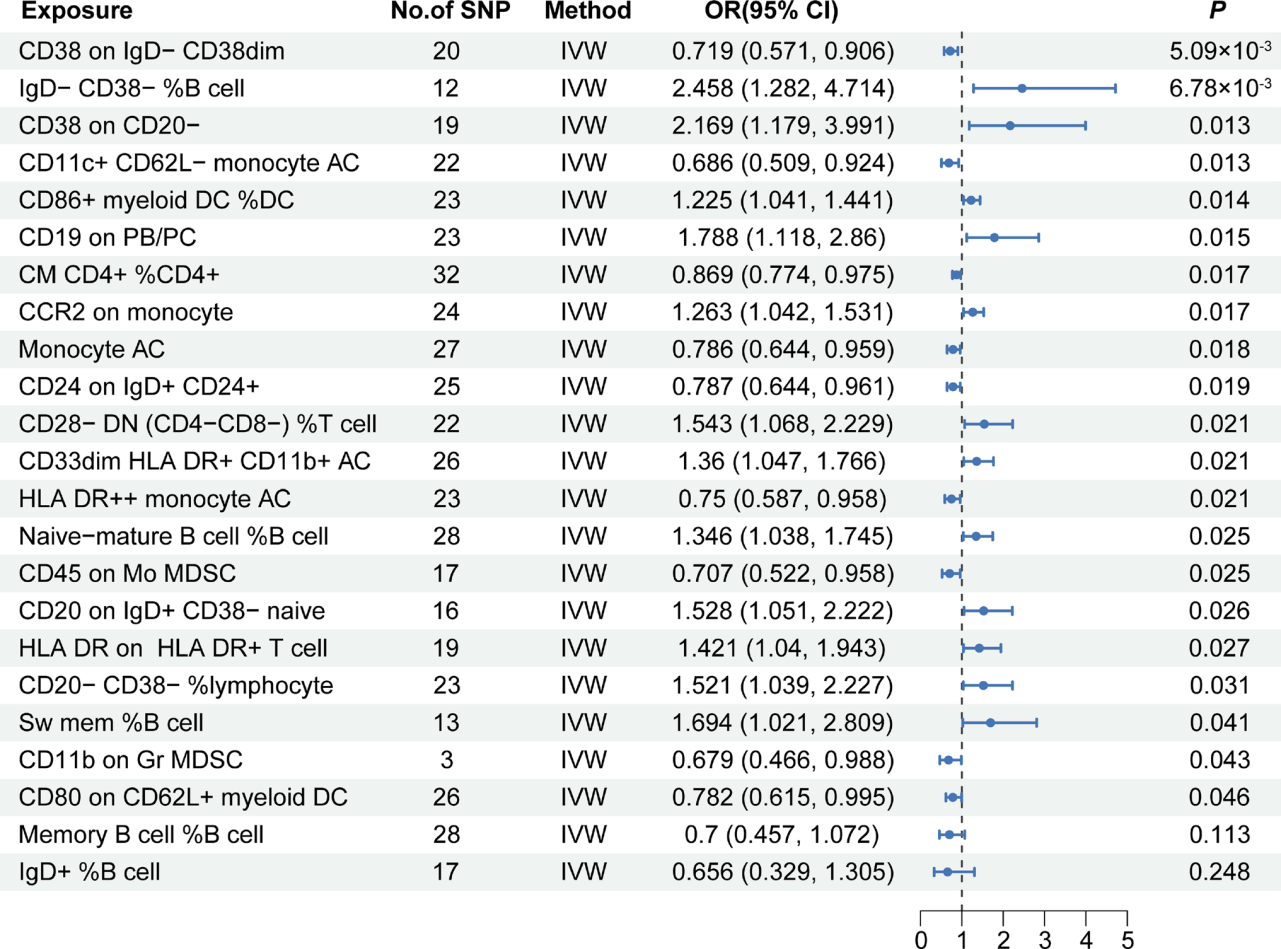
Forest plot of causal effects for immune traits on CHS. CHS, chondrosarcoma; IVW, inverse-variance weighted

**Fig. 3:**
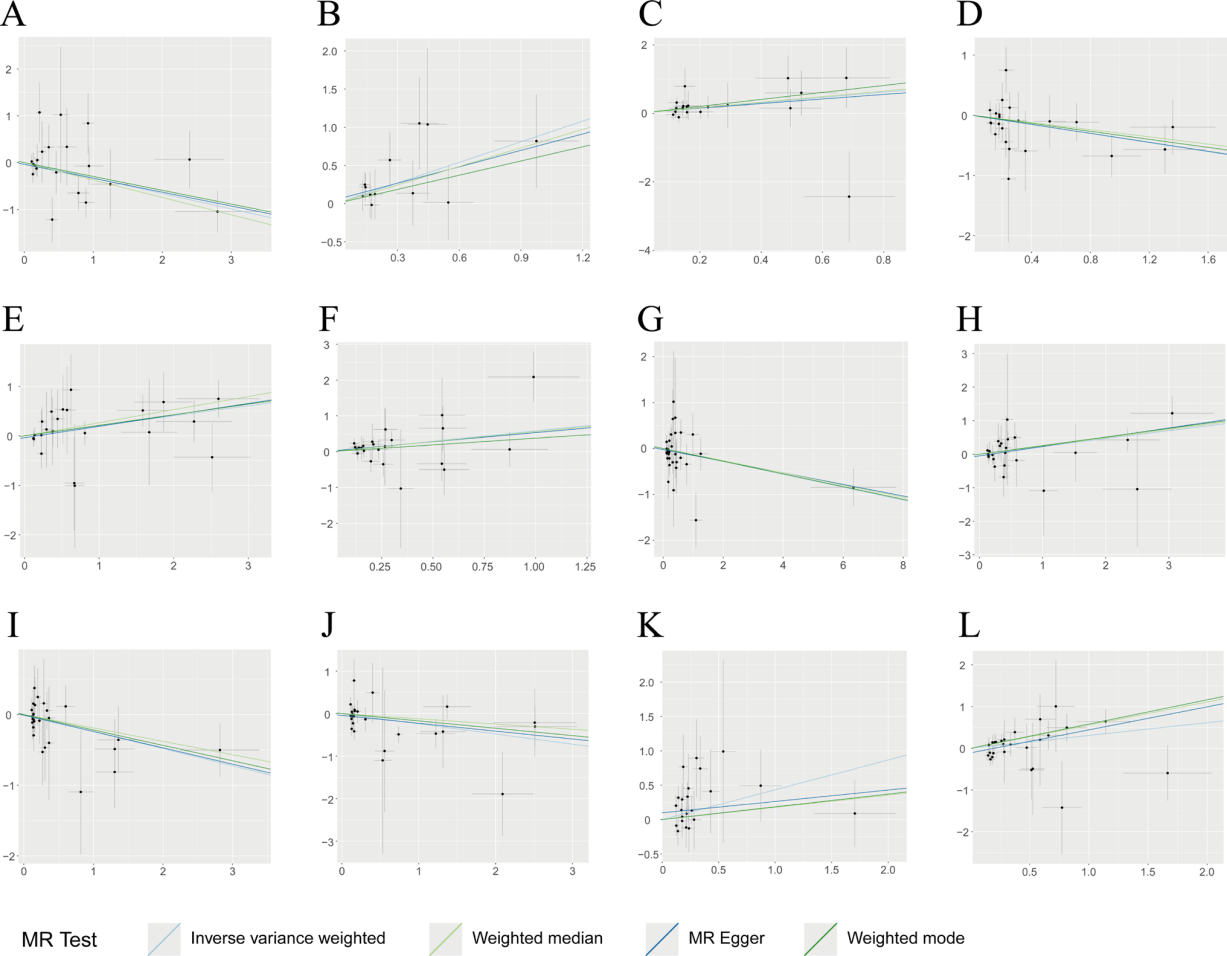
Scatter plot of causal effects for immune traits on CHS. **A** CD38 on IgD- CD38dim on CHS, **B** IgD- CD38- %B cell on CHS, **C** CD38 on CD20- on CHS, **D** CD11c + CD62L- monocyte AC on CHS, **E** CD86 + myeloid DC %DC on CHS, **F** CD19 on PB/PC on CHS, **G** CM CD4 + %CD4 + on CHS, **H** CCR2 on monocyte on CHS, **I** Monocyte AC on CHS, **J** CD24 on IgD + CD24 + on CHS, **K** CD28- DN (CD4-CD8-) %T cell on CHS, **L** CD33dim HLA DR + CD11b + AC on CHS

**Fig. 4:**
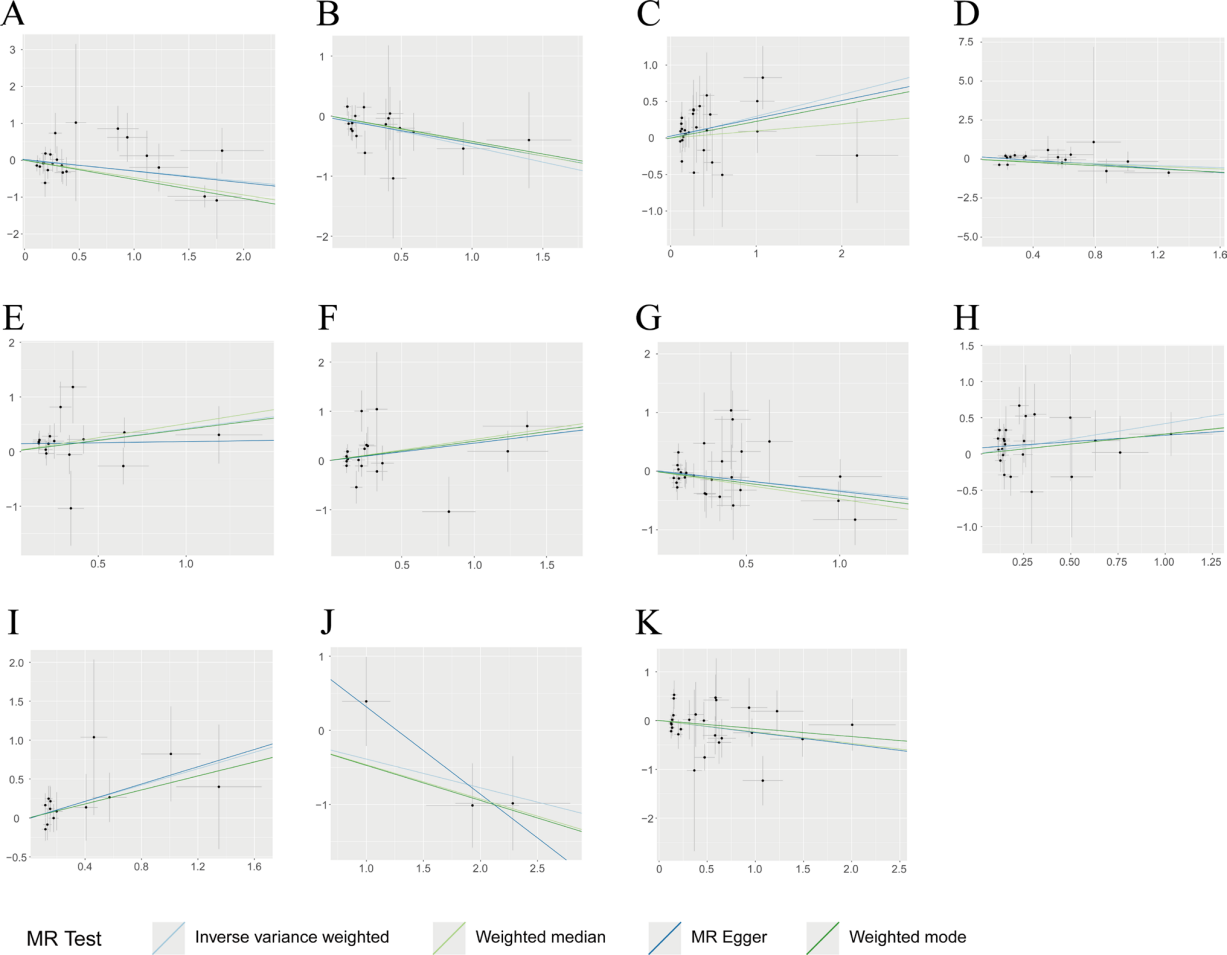
Scatter plot of causal effects for immune traits on CHS. **A** HLA DR +  + monocyte AC on CHS, **B** IgD + %B cell on CHS, **C** Naive-mature B cell %B cell on CHS, **D** CD45 on Mo MDSC on CHS, **E** CD20 on IgD + CD38- naïve on CHS, **F** HLA DR on HLA DR + T cell on CHS, **G** Memory B cell %B cell on CHS, **H** CD20- CD38- %lymphocyte on CHS, **I** Sw mem %B cell on CHS, **J** CD11b on Gr MDSC on CHS, **K** CD80 on CD62L + myeloid DC on CHS

The results of the MR analysis suggested that 23 immune traits were associated with CHS, including 11 in the B cell panel, 3 in the cDC, 1 in the maturation stages of the T cell panel, 2 in the monocyte panel, 3 in the myeloid cell panel, 2 in the TBNK panel, and 1 in the Treg panel. Among these immune traits, elevated levels of 12 immune traits were associated with a high risk of CHS (OR > 1, *P* < 0.05), of which 7 belonged to the B cell panel (IgD- CD38- %B cell, CD38 on CD20-, CD19 on PB/PC, Naive-mature B cell %B cell, CD20 on IgD + CD38- naïve, CD20- CD38- %lymphocyte, Sw mem %B cell), 1 to the cDC panel (CD86 + myeloid DC %DC), 1 to the Monocyte panel (CCR2 on monocyte), 1 to the Myeloid cell panel (CD33dim HLA DR + CD11b + AC), 1 to the TBNK panel (HLA DR on HLA DR + T cell), and 1 to the Treg panel (CD28- DN (CD4-CD8-) %T cell), while the other 11 immune traits were detected as potential protective factors for CHS (OR < 1, *P* < 0.05), of which 4 belonged to B cell panel (CD38 on IgD- CD38dim, CD24 on IgD + CD24 + , IgD + %B cell, Memory B cell %B cell), 2 belonged to cDC panel (CD11c + CD62L- monocyte AC, CD80 on CD62L + myeloid DC), 1 belonged to Maturation stages of T cell panel (CM CD4 + %CD4 +), 1 belonged to Monocyte panel (Monocyte AC), 2 belonged to Myeloid cell panel (CD45 on Mo MDSC, CD11b on Gr MDSC), and 1 belonged to TBNK panel (HLA DR +  + monocyte AC). No significant heterogeneity or horizontal pleiotropy was observed in this study. Further details regarding the results of MR and sensitivity analyses are presented in Supplementary Table 2. MR Steiger testing did not indicate any evidence of reverse causation. Information on the IVs, MR Steiger test, and *F*-statistic are presented in Supplementary Table 3. Figure 2 shows the forest plot, and scatter plots are shown in Fig. 3–4. The funnel plots are shown in Supplementary Fig. 1, 2 and the plots of the leave-one-out analysis are presented in Supplementary Fig. 3, 4.

### The causal relationship between inflammatory proteins and CHS

**Fig. 5:**
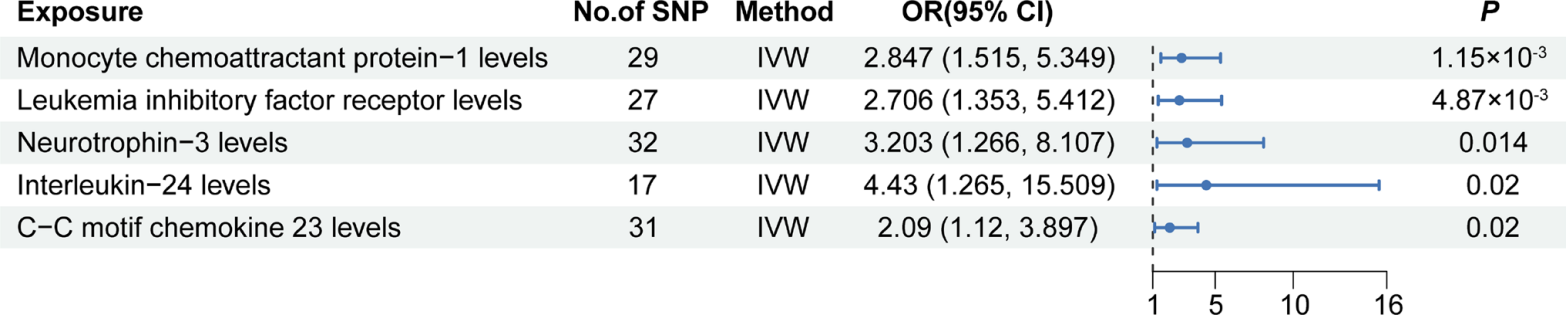
Forest plot of causal effects for inflammatory proteins on CHS

**Fig. 6:**
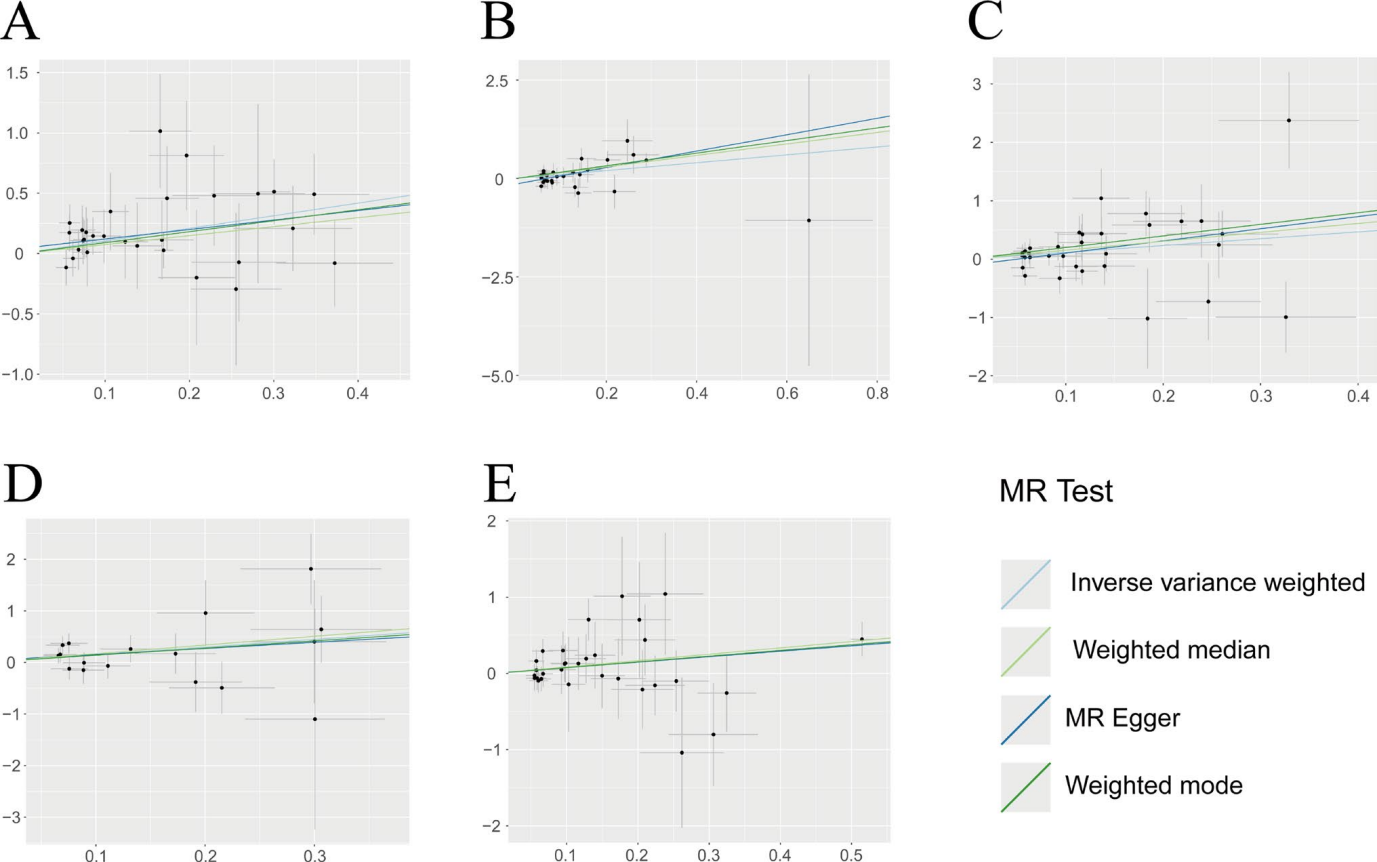
Scatter plot of causal effects for inflammatory proteins on CHS. **A** Monocyte chemoattractant protein-1 levels on CHS, **B** Leukemia inhibitory factor receptor levels on CHS, **C** Neurotrophin-3 levels on CHS, **D** Interleukin-24 levels on CHS, **E** C–C motif chemokine 23 levels on CHS

Our results showed that five circulating inflammatory proteins (monocyte chemoattractant protein-1, leukemia inhibitory factor receptor levels, Neurotrophin-3 levels, IL-24, and C–C motif chemokine 23) were the risk factors for CHS (OR > 1, P < 0.05). The forest plot is shown in Fig. 5, and the scatter plot is shown in Fig. 6. Funnel plots and leave-one-out analyses are shown in Supplementary Fig. 5 and Supplementary Fig. 6, respectively. The MR-Egger intercept and MR-PRESSO tests did not reveal significant horizontal pleiotropy. Cochran's Q test did not detect significant heterogeneity. MR Steiger test found no evidence of reverse causation. The results of the causal inference and sensitivity analysis are presented in Supplementary Table 4, while the detailed information on the IVs is presented in Supplementary Table 5.

### Mediation analysis

We evaluated the causal relationship between the screened exposures, based on nominally significant results for immune traits and inflammatory proteins in CHS. Ultimately, five causal relationships were identified between CHS-associated immune traits and CHS-associated inflammatory proteins. Further details are provided in Supplementary Table 6. Nevertheless, the results of the mediation analysis did not indicate significant mediating effects (Supplementary Table 7).

## Discussion

The causal relationships between CHS and 731 immune traits, as well as 91 inflammatory factors, were assessed using a two-sample MR analysis based on publicly available GWAS summary data. A total of 23 immune traits were identified as having a causal effect on CHS. Elevated levels of five inflammatory proteins were associated with an increased risk of CHS. No evidence of pleiotropy or heterogeneity was observed in this study. The MR-Steiger directional analysis confirmed the hypothesized causal directionality, effectively distinguishing exposure-outcome relationships from reverse causation pathways. The results of the mediation analysis indicated that inflammatory proteins did not mediate the relationships between the immune traits and CHS.

### Exploration of immunotherapy for CHS

Malignant tumors arise from genomic instability, including chromosomal rearrangements and epigenetic alterations, which lead to the activation of oncogenes and the loss of function of tumor suppressors. These tumors are characterized by a high proliferation rate, invasiveness, propensity for metastasis, and poor prognosis [30]. The environment in which tumor cells reside is referred to as the TME. It comprises a diverse array of cells, including immune traits, tumor cells, and fibroblasts, as well as a variety of cytokines, chemokines, and tumor stromal components [31]. The concept of “tumor immune microenvironment (TIME)” was introduced by Krummel et al. in 2018. They suggested that increasing the infiltration of cytotoxic T-lymphocytes in TIME is crucial for improving the efficacy of immune checkpoint inhibitors (ICIs), such as PD-1, programmed death ligand-1 (PD-L1) monoclonal antibody, and cytotoxic T-lymphocyte-associated protein-4 (CTLA-4) [32]. Chondrosarcoma is a common malignancy for which surgical intervention is considered beneficial. However, due to its insensitivity to both radiotherapy and chemotherapy, exploring alternative treatment strategies is urgently needed for patients who are not candidates for surgery or who experience recurrent metastases following surgical treatment. The field of immuno-oncology is dedicated to elucidating the mechanisms underlying evasion of immune detection and destruction, as well as to controlling and eliminating cancer by reactivating and maintaining the immune system’s ability to recognize and eliminate tumor cells [33]. Several studies have explored the potential of immunotherapy for CHS. Vascular endothelial growth factor A (VEGF-A), secreted by tumor cells, binds to the transmembrane tyrosine kinase receptors VEGFR-1 and VEGFR-2 on endothelial cells, thereby inducing angiogenesis in solid tumors. This process enhances tumor cell survival, migration, and proliferation. Pazopanib is a multitargeted tyrosine kinase inhibitor that targets the VEGF receptor, platelet-derived growth factor (PDGF) receptor. A phase 2 clinical trial reported its efficacy in unresectable and metastatic conventional CHS, with 43% of patients achieving partial remission or stable disease and a median overall survival of 17.6 months [34]. Similarly, regorafenib, a VEGF receptor-targeted agent, was found to prolong progression-free survival in patients with advanced CHS [35]. There is a correlation between the composition of the immune infiltrate, response to immunotherapy, and survival in CHS. The efficacy of the anti-PD-1 antibody pembrolizumab was investigated in a Phase II clinical trial involving five patients with advanced CHS. Among them, one patient achieved an objective response, one had stable disease, and three experienced progressive disease [36]. Among 12 CHS patients treated with anti-PD-1 antibodies, only three patients with partial remission or stable disease were identified as having the “immune-depleted” subtype, characterized by very low infiltration of monocytes, macrophages, B-cells, and NK-cells [14]. These reports highlight the importance of further exploring and defining immune traits and subtypes to guide stratification and treatment strategies for patients with CHS.

### Our findings and available evidence

Cluster of Differentiation 38 (CD38) was initially identified in 1980 by Reinherz et al. Since then, it has been shown to be widely expressed across a diverse range of immune cells, including B lymphocytes [37]. CD38 is a multifunctional protein that is primarily believed to be a type II transmembrane glycoprotein, characterized by its exoenzyme activity [38]. It belongs to the NAD + -hydrolyzed enzymes/5-diphosphate adenosine ribonucleic cyclase family and is involved in regulating immune cell activation, maturation, differentiation, and immune tolerance [39]. In addition, CD38 is located on human chromosome 4 and mouse chromosome 5, exhibiting approximately 70% sequence homology between the two species [40]. Notably, there are significant differences between human and murine CD38 in terms of tissue distribution and functions [39]. The protumorigenic function of CD38 in hematologic malignancies has been extensively investigated. Daratumumab, the first antibody targeting CD38, has demonstrated sustained anti-tumor responses in clinical settings through mechanisms including antibody-dependent cell-mediated cytotoxicity, antibody-dependent cellular phagocytosis, and complement-dependent cytotoxicity [41]. It has been used to treat relapsed and refractory multiple myeloma. Several studies have documented the progression of CD38-targeting antibodies in other hematologic neoplasms, including chronic lymphocytic leukemia, T-cell acute lymphoblastic leukemia, and natural killer (NK)/T-cell lymphoma [42–44]. Nevertheless, the role of CD38 in solid tumors remains largely unexplored [45]. Available evidence suggests that CD38 is predominantly identified as a pro-carcinogenic factor, although some conflicting findings have been reported. Levy et al. demonstrated that CD38 deficiency led to the downregulation of tumor-associated microglia and macrophage infiltration, a notable increase in tumor cell mortality, and a significant attenuation of glioma development. Additionally, the lifespan of CD38 knockout mice was prolonged [46]. The expression of CD38 RNA and protein was higher in cervical cancer tissues compared to normal tissues [47]. In esophageal cancer, the accumulation of inflammation-derived secretory factors, including IL-6, Insulin-like growth factor-binding protein-3 (IGFBP-3), and CXC motif chemokine ligand 16 (CXCL16), results in the upregulation of CD38 in myeloid-derived suppressor cells (MDSCs) [48]. CD38 + MDSCs exhibit an immature state of differentiation and possess a greater capacity to inhibit intra-tumor T-cell activity, thereby promoting tumor progression. The implantation of MDSCs crosslinked with anti-CD38 antibodies has been shown to significantly inhibit tumor growth [48]. However, conflicting findings have been reported regarding CD38 expression in prostate cancer. Guo et al. discovered that the density of filtering immune cells within CD38 + tumors is a predictor of progression in castration-resistant prostate cancer and is associated with a poorer prognosis [49]. Additionally, multiplexed immunofluorescence results indicated that the density of CD38 + tumor-infiltrating immune cells is associated with reduced survival and immunosuppressive mechanisms in the tumor microenvironment [49]. In contrast, another study demonstrated that CD38 expression is negatively correlated with tumor progression in prostate cancer [50]. Overexpression of CD38 in prostate cancer cells leads to an increase in pAMPK levels, a decrease in fatty acid and lipid synthesis, reduced glycolysis and metabolism, and an extended cell doubling time, ultimately resulting in a decrease in tumor cell proliferation [50]. Since CD38 expression results in deficient fatty acid and lipid synthesis, this may be detrimental to the adipogenic phenotype of prostate cancer. Thus, the function of CD38 may vary depending on the metabolic requirements of different solid tumors. Similarly, CD38 has been identified as an independent prognostic factor in patients with triple-negative breast cancer, where higher expression of CD38 on tumor-infiltrating plasma cells is associated with increased metastasis-free survival and overall survival [51]. In hepatocellular carcinoma (HCC), high density of CD38 + macrophage was associated with improved survival [52]. A higher number of CD38 + cells in the tumor was strongly associated with a favorable response to Immune-checkpoint blockade in HCC, implying longer median progression-free survival and median overall survival [53]. Our findings suggest that more CD38 on IgD- CD38dim is associated with lower CHS risk, and increases in IgD- CD38- %B cell and CD38 on CD20- suggest higher CHS risk. Differences in CD38 expression across various B cell phenotypes may exert distinct effects on CHS, and these findings warrant further validation through additional studies. To date, no studies have specifically examined CD38 expression in CHS. Further investigation is required to gain a deeper understanding of the infiltrating immune populations in CHS that express and utilize CD38, their interactions with tumor cells, and the potential targeting of these cells and molecules for the effective elimination of CHS.

The findings of our study indicate a significant correlation between the B-cell panel and the progression of CHS. B cells can be activated through T-cell-dependent (TD) or T-cell-independent (TI) manner, undergo proliferation, and differentiate into plasmablasts (PBs) [54]. The differentiation of B cells into either immunoglobulin antibody-producing plasma cells (PCs) or memory B cells is dependent on the specific activation conditions [55]. It is common practice to identify major B cell populations in peripheral blood based on the expression of specific B cell surface molecules, including IgD, CD19, CD20, CD27, CD38, and CD138 [56]. Our results suggest that CD19 on PB/PC has a positive causal effect on CHS. In vitro studies have demonstrated that targeting CD38 with dalimumab or knocking down CD38 inhibits the binding of CD19 to the BCR, interferes with B-cell activation in cell lines, suppresses B-cell proliferation and differentiation into PBs and PCs, and correlates with the attenuation of BCR signaling in both normal and malignant human B-cell lines [57]. Indeed, B cell maturation is associated with the involvement of CD38 [58]. Furthermore, treatment with dalimumab resulted in a change in the composition of T cells, with a notable increase in the proportion of HLA-DR-positive CD8 + T cells [59]. HLA-DR-specific monocytes collected from a patient with CHS spontaneously differentiated into neoplastic fibroblasts that exhibited persistent and abnormal proliferation [60]. Mechanistic investigations revealed that infiltration of HLA-DR + monocyte within necrotic areas and their subsequent transdifferentiation into tumor-promoting fibroblasts and pseudo-chondrocytes may drive CHS progression through stromal remodeling [60]. Minopoli et al. proposed that inhibiting monocyte recruitment and infiltration into CHS can attenuate the pro-tumorigenic activity of tumor-associated macrophages in CHS [61]. These findings are inconsistent with our results, highlighting the need for further research to elucidate the role of monocytes and their sub-populations in CHS. Our results showed a positive causal relationship between monocyte chemoattractant protein-1 (MCP-1) levels and CHS progression. MCP-1 (chemokine ligand 2/CCL2), a pivotal member of the CC chemokine superfamily, orchestrates tumor-stromal interactions by dually modulating neoplastic proliferation and angiogenic programming. Its pro-metastatic activity has been clinically validated in various malignancies, including breast, prostate and colorectal carcinomas [62–64]. Furthermore, Tang's findings indicated that MCP-1 enhances MMP-9 expression and cell migration in human CHS cells [65]. To some extent, these studies support our results. Furthermore, our results suggest that inflammatory proteins do not mediate the causal effect of immune traits on CHS. A possible reason is that the interactions between immune traits and inflammatory proteins are highly diverse and complex. Our study included only 731 immune traits and 91 inflammatory proteins, and many immune factors were not evaluated. Moreover, the limited sample size may have restricted our ability to detect mediating effects.

### Clinical reflections on this study

Our study provides insights into the stratification of immunotherapy for CHS patients. High expression of certain immune traits or inflammatory proteins may be associated with faster progression of CHS or greater sensitivity to specific treatments. For example, our study suggests that higher levels of MCP-1 may indicate active CHS. Therefore, the study and application of cytokine inhibitors targeting MCP-1 may hold potential value in this patient population [66]. By determining the immune microenvironment of tumor tissues from CHS patients, the application of specific immune checkpoint inhibitors or cytokine inhibitors tailored to different immune traits may better achieve the clinical goals. For example, our results suggest that certain immune traits in the B-cell panel significantly contribute to CHS progression. Therefore, for CHS populations with prominent CD19 expression on PB/PC, inhibiting the interaction between CD19 and PB/PC may better achieve the goals of precision and personalized immunotherapy. The search for novel tumor markers, including biomarkers and genetic factors that can predict response to immunotherapy or identify new targets that determinetumor malignancy, holds great value. It will facilitate the development of new immunotherapeutic treatments, improve the interpretation and assessment of patient response, and create opportunities for personalized treatment strategies [67, 68]. The integration of individual oncogenic and immune trait, combined with the identification of biomarkers, enables precision medicine in oncology. Personalized immunotherapy, which takes these factors into account, offers a promising avenue for improving outcomes in cancer patients [69].

### Future prospects

Cancer immunotherapy harnesses the body's immune system to detect and destroy cancer cells more efficiently and selectively. It has achieved remarkable success in treating and controlling tumors, emerging as a major focus in cancer research and therapy [70]. However, tumors may create an immunosuppressive microenvironment through the presence of immunosuppressive cells and the secretion of molecules that impede the infiltration of immune traits and immunomodulators, or induce immune trait dysfunction. Additionally, overcoming immune-related adverse effects and achieving a balance in the regulation of immune responses remains a significant challenge. Researchers are utilizing intelligent drug delivery systems to co-deliver multiple therapeutic agents directly to tumor cells or immune-suppressing cells, thereby increasing drug concentrations at the target site and improving efficacy [71]. Nanoparticles are a class of natural or artificial materials with nanoscale dimensions that have the potential to influence the function of immune cells, including macrophages, dendritic cells, T lymphocytes, and NK cells. This modulation can be achieved through various mechanisms, such as enhancing cytokine production, improving antigen presentation, boosting T-cell responses, and overcoming immunosuppressive tumor environments [72]. The development of emerging multifunctional nanoparticles is likely to offer new and enhanced approaches for the precision and effectiveness of cancer diagnosis and treatment.

## Limitations

Several considerations warrant cautious interpretation of our findings. Firstly, it should be acknowledged that the findings of this study have limited applicability to populations other than those of European origin. Second, the available GWAS data for CHS do not provide information on CHS subtypes, thereby limiting our ability to elucidate the specific subtypes of CHS. We look forward to larger and more detailed GWAS data on CHS being published in the future to support further studies.

## Conclusion

In summary, we explored the causal relationship between immune factors and CHS using a comprehensive two-sample MR analysis, which is the first attempt in this area using MR. These findings deepen our understanding of the immune mechanisms underlying CHS and provide valuable insights for the study of CHS immunotherapy. Our study lays the foundation for evaluating potential biomarkers for CHS diagnosis, treatment, and prognosis, thereby contributing to the further exploration of early interventions in CHS.

## Supplementary Information

Supplementary file 1. Fig. 1 Funnel plot of potential causality of immune traits on CHS. (A) CD38 on IgD- CD38dim on CHS, (B) IgD- CD38- %B cell on CHS, (C) CD38 on CD20- on CHS, (D) CD11c+ CD62L- monocyte AC on CHS, (E) CD86+ myeloid DC %DC on CHS, (F) CD19 on PB/PC on CHS, (G) CM CD4+ %CD4+ on CHS, (H) CCR2 on monocyte on CHS, (I) Monocyte AC on CHS, (J) CD24 on IgD+ CD24+ on CHS, (K) CD28- DN (CD4-CD8-) %T cell on CHS, (L) CD33dim HLA DR+ CD11b+ AC on CHS. Fig. 2 Funnel plot of potential causality of immune traits on CHS. (A) HLA DR++ monocyte AC on CHS, (B) IgD+ %B cell on CHS, (C) Naive-mature B cell %B cell on CHS, (D) CD45 on Mo MDSC on CHS, (E) CD20 on IgD+ CD38- naïve on CHS, (F) HLA DR on HLA DR+ T cell on CHS, (G) Memory B cell %B cell on CHS, (H) CD20- CD38- %lymphocyte on CHS, (I) Sw mem %B cell on CHS, (J) CD11b on Gr MDSC on CHS, (K) CD80 on CD62L+ myeloid DC on CHS. Fig. 3 Leave-one-out sensitivity analysis of potential causality of immune traits on CHS. (A) CD38 on IgD- CD38dim on CHS, (B) IgD- CD38- %B cell on CHS, (C) CD38 on CD20- on CHS, (D) CD11c+ CD62L- monocyte AC on CHS, (E) CD86+ myeloid DC %DC on CHS, (F) CD19 on PB/PC on CHS, (G) CM CD4+ %CD4+ on CHS, (H) CCR2 on monocyte on CHS, (I) Monocyte AC on CHS, (J) CD24 on IgD+ CD24+ on CHS, (K) CD28- DN (CD4-CD8-) %T cell on CHS, (L) CD33dim HLA DR+ CD11b+ AC on CHS. Fig. 4 Leave-one-out sensitivity analysis of potential causality of immune traits on CHS. (A) HLA DR++ monocyte AC on CHS, (B) IgD+ %B cell on CHS, (C) Naive-mature B cell %B cell on CHS, (D) CD45 on Mo MDSC on CHS, (E) CD20 on IgD+ CD38- naïve on CHS, (F) HLA DR on HLA DR+ T cell on CHS, (G) Memory B cell %B cell on CHS, (H) CD20- CD38- %lymphocyte on CHS, (I) Sw mem %B cell on CHS, (J) CD11b on Gr MDSC on CHS, (K) CD80 on CD62L+ myeloid DC on CHS. Fig. 5 Funnel plot of potential causality of inflammatory protein on CHS. (A) Monocyte chemoattractant protein-1 levels on CHS, (B) Leukemia inhibitory factor receptor levels on CHS, (C) Neurotrophin-3 levels on CHS, (D) Interleukin-24 levels on CHS, (E) C-C motif chemokine 23 levels on CHS. Fig. 6 Leave-one-out sensitivity analysis of potential causality of inflammatory protein on CHS. (A) Monocyte chemoattractant protein-1 levels on CHS, (B) Leukemia inhibitory factor receptor levels on CHS, (C) Neurotrophin-3 levels on CHS, (D) Interleukin-24 levels on CHS, (E) C-C motif chemokine 23 levels on CHS.

Supplementary file 2. Table 1. STROBE-MR checklist of recommended items to address in reports of Mendelian randomization studies

Supplementary file 3. Table 2. Results of the causal effect of immune traits on chondrosarcoma. Table 3. The instrumental variables for significant immune traits used in MR analyses of immune traits on chondrosarcoma. Table 4. Results of the causal effect of inflammatory proteins on chondrosarcoma. Table 5. The instrumental variables for the significant inflammatory protein used in MR analyses of inflammatory proteins on chondrosarcoma. Table 6. Causal effects of immune traits associated with chondrosarcoma on inflammatory proteins related to chondrosarcoma. Table 7. The mediation effect of immune traits on chondrosarcoma via inflammatory proteins

## Data Availability

The original contributions presented in the study are included in the article/supplementary material, further inquiries can be directed to the corresponding author.
