# Supplementary material for: Exploring the causal relationship between immune factors and chondrosarcoma: a Mendelian randomization study
Source: Discov Oncol. 2025 May 18;16:801. doi: 10.1007/s12672-025-02654-5 (PMC12086138; doi:10.1007/s12672-025-02654-5)
Supplement: Supplementary file 1 — Fig. 1 Funnel plot of potential causality of immune traits on CHS. (A) CD38 on IgD- CD38dim on CHS, (B) IgD- CD38- %B cell on CHS, (C) CD38 on CD20- on CHS, (D) CD11c+ CD62L- monocyte AC on CHS, (E) CD86+ myeloid DC %DC on CHS, (F) CD19 on PB/PC on CHS, (G) CM CD4+ %CD4+ on CHS, (H) CCR2 on monocyte on CHS, (I) Monocyte AC on CHS, (J) CD24 on IgD+ CD24+ on CHS, (K) CD28- DN (CD4-CD8-) %T cell on CHS, (L) CD33dim HLA DR+ CD11b+ AC on CHS. Fig. 2 Funnel plot of potential causality of immune traits on CHS. (A) HLA DR++ monocyte AC on CHS, (B) IgD+ %B cell on CHS, (C) Naive-mature B cell %B cell on CHS, (D) CD45 on Mo MDSC on CHS, (E) CD20 on IgD+ CD38- naïve on CHS, (F) HLA DR on HLA DR+ T cell on CHS, (G) Memory B cell %B cell on CHS, (H) CD20- CD38- %lymphocyte on CHS, (I) Sw mem %B cell on CHS, (J) CD11b on Gr MDSC on CHS, (K) CD80 on CD62L+ myeloid DC on CHS. Fig. 3 Leave-one-out sensitivity analysis of potential causality of immune traits on CHS. (A) CD38 on IgD- CD38dim on CHS, (B) IgD- CD38- %B cell on CHS, (C) CD38 on CD20- on CHS, (D) CD11c+ CD62L- monocyte AC on CHS, (E) CD86+ myeloid DC %DC on CHS, (F) CD19 on PB/PC on CHS, (G) CM CD4+ %CD4+ on CHS, (H) CCR2 on monocyte on CHS, (I) Monocyte AC on CHS, (J) CD24 on IgD+ CD24+ on CHS, (K) CD28- DN (CD4-CD8-) %T cell on CHS, (L) CD33dim HLA DR+ CD11b+ AC on CHS. Fig. 4 Leave-one-out sensitivity analysis of potential causality of immune traits on CHS. (A) HLA DR++ monocyte AC on CHS, (B) IgD+ %B cell on CHS, (C) Naive-mature B cell %B cell on CHS, (D) CD45 on Mo MDSC on CHS, (E) CD20 on IgD+ CD38- naïve on CHS, (F) HLA DR on HLA DR+ T cell on CHS, (G) Memory B cell %B cell on CHS, (H) CD20- CD38- %lymphocyte on CHS, (I) Sw mem %B cell on CHS, (J) CD11b on Gr MDSC on CHS, (K) CD80 on CD62L+ myeloid DC on CHS. Fig. 5 Funnel plot of potential causality of inflammatory protein on CHS. (A) Monocyte chemoattractant protein-1 levels on CHS, (B) Leukemia inhibitory factor receptor levels on CHS, (C) [file 12672_2025_2654_MOESM1_ESM.docx]

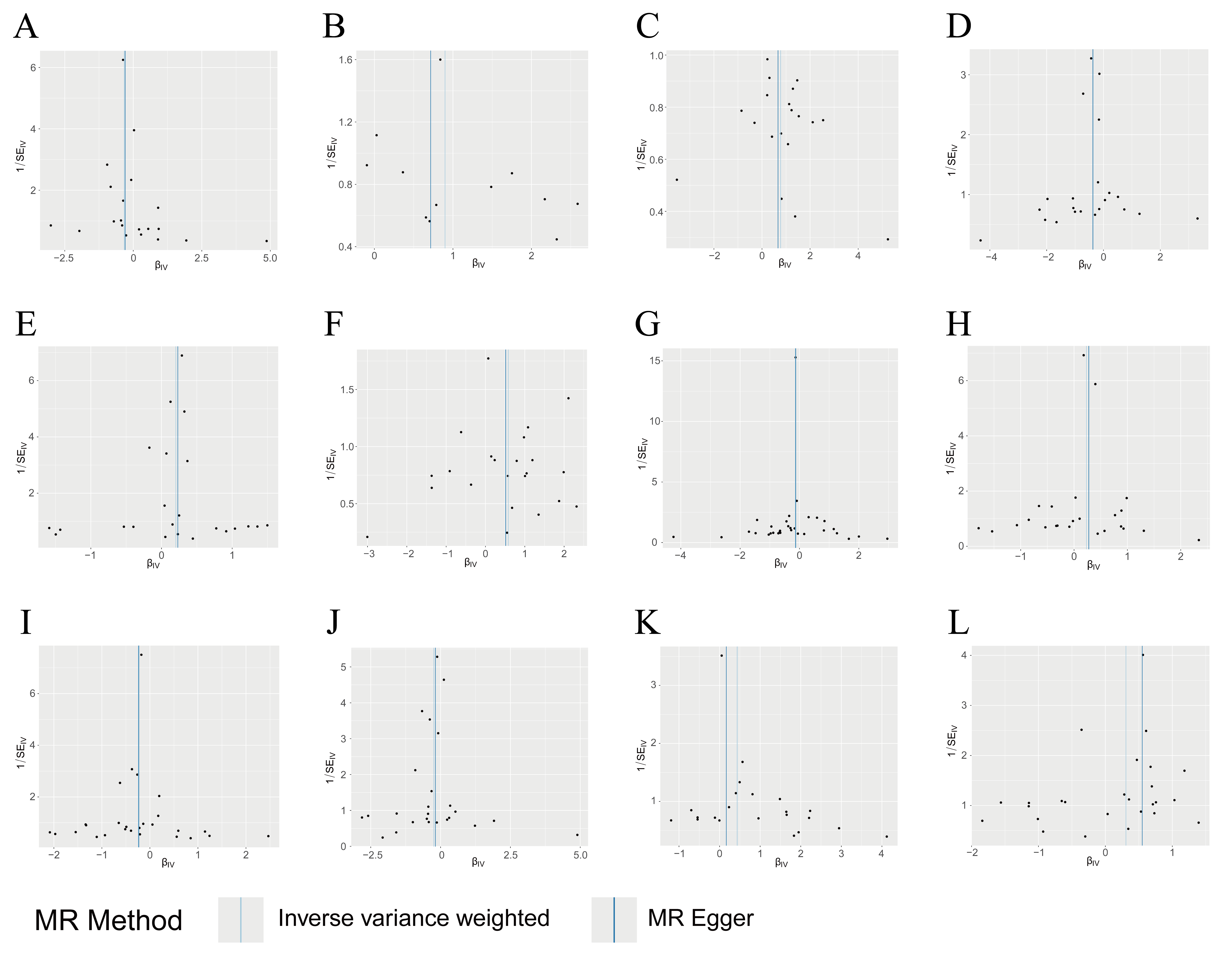


**Supplementary Fig. 1** Funnel plot of potential causality of immune cells on CHS. **(A)** CD38 on IgD- CD38dim on CHS, **(B)** IgD- CD38- %B cell on CHS, **(C)** CD38 on CD20- on CHS, **(D)** CD11c+ CD62L- monocyte AC on CHS, **(E)** CD86+ myeloid DC %DC on CHS, **(F)** CD19 on PB/PC on CHS, **(G)** CM CD4+ %CD4+ on CHS, **(H)** CCR2 on monocyte on CHS, **(I)** Monocyte AC on CHS, **(J)** CD24 on IgD+ CD24+ on CHS, **(K)** CD28- DN (CD4-CD8-) %T cell on CHS, **(L)** CD33dim HLA DR+ CD11b+ AC on CHS.


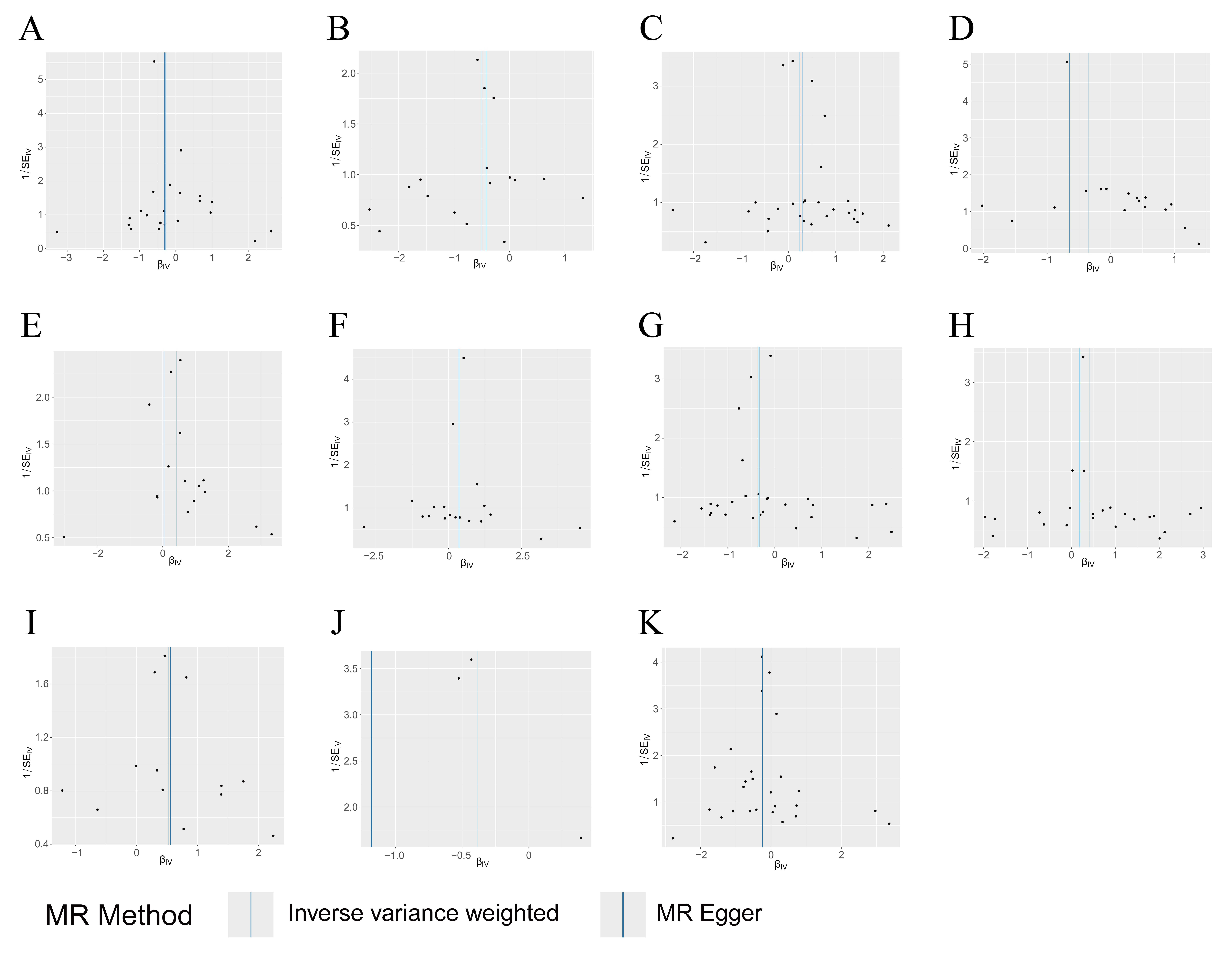


**Supplementary Fig. 2** Funnel plot of potential causality of immune cells on CHS. **(A)** HLA DR++ monocyte AC on CHS, **(B)** IgD+ %B cell on CHS, **(C)** Naive-mature B cell %B cell on CHS, **(D)** CD45 on Mo MDSC on CHS, **(E)** CD20 on IgD+ CD38- naïve on CHS, **(F)** HLA DR on HLA DR+ T cell on CHS, **(G)** Memory B cell %B cell on CHS, **(H)** CD20- CD38- %lymphocyte on CHS, **(I)** Sw mem %B cell on CHS, **(J)** CD11b on Gr MDSC on CHS, **(K)** CD80 on CD62L+ myeloid DC on CHS.


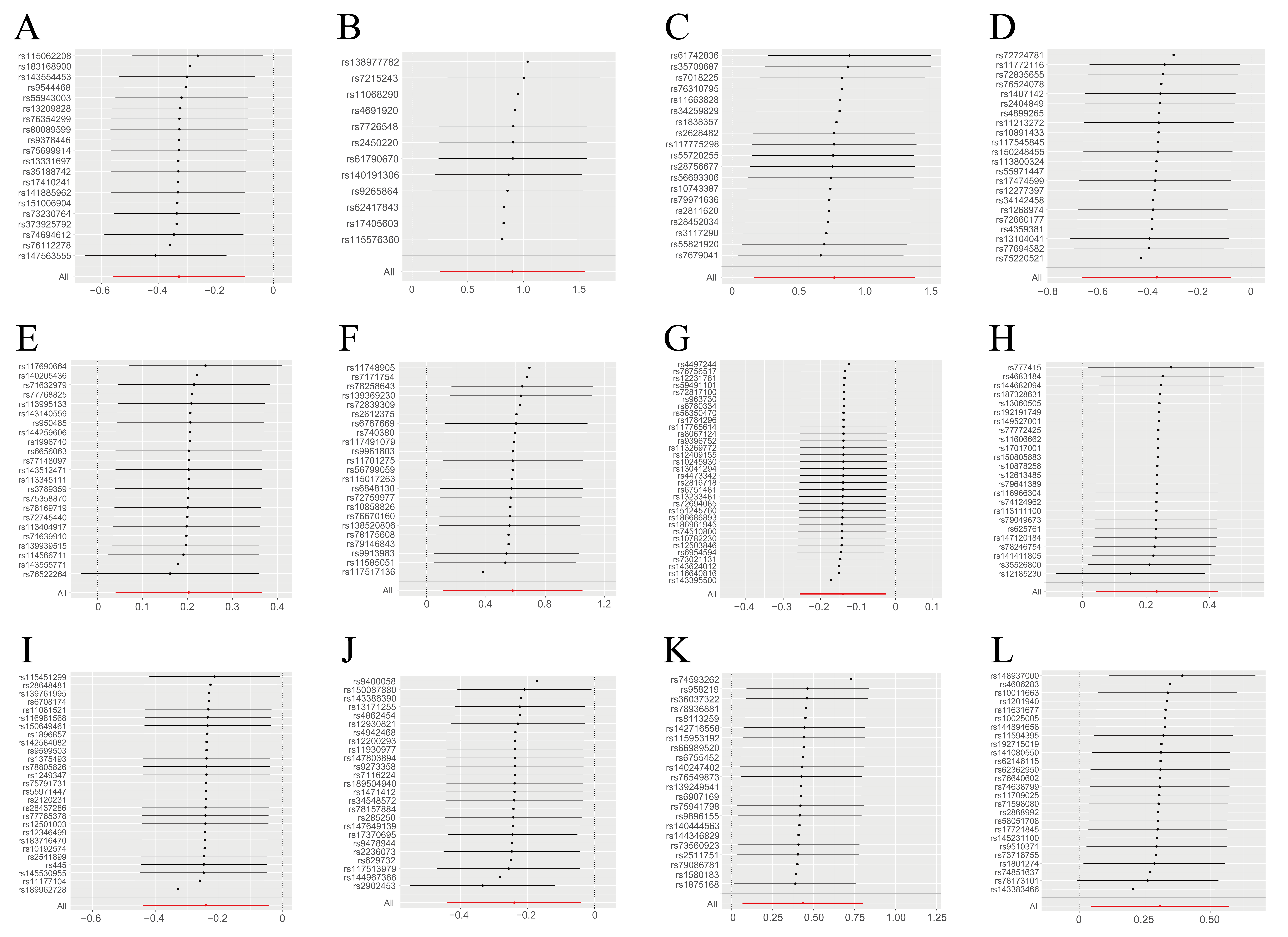


**Supplementary Fig. 3** Leave-one-out sensitivity analysis of potential causality of immune cells on CHS. **(A)** CD38 on IgD- CD38dim on CHS, **(B)** IgD- CD38- %B cell on CHS, **(C)** CD38 on CD20- on CHS, **(D)** CD11c+ CD62L- monocyte AC on CHS, **(E)** CD86+ myeloid DC %DC on CHS, **(F)** CD19 on PB/PC on CHS, **(G)** CM CD4+ %CD4+ on CHS, **(H)** CCR2 on monocyte on CHS, **(I)** Monocyte AC on CHS, **(J)** CD24 on IgD+ CD24+ on CHS, **(K)** CD28- DN (CD4-CD8-) %T cell on CHS, **(L)** CD33dim HLA DR+ CD11b+ AC on CHS.


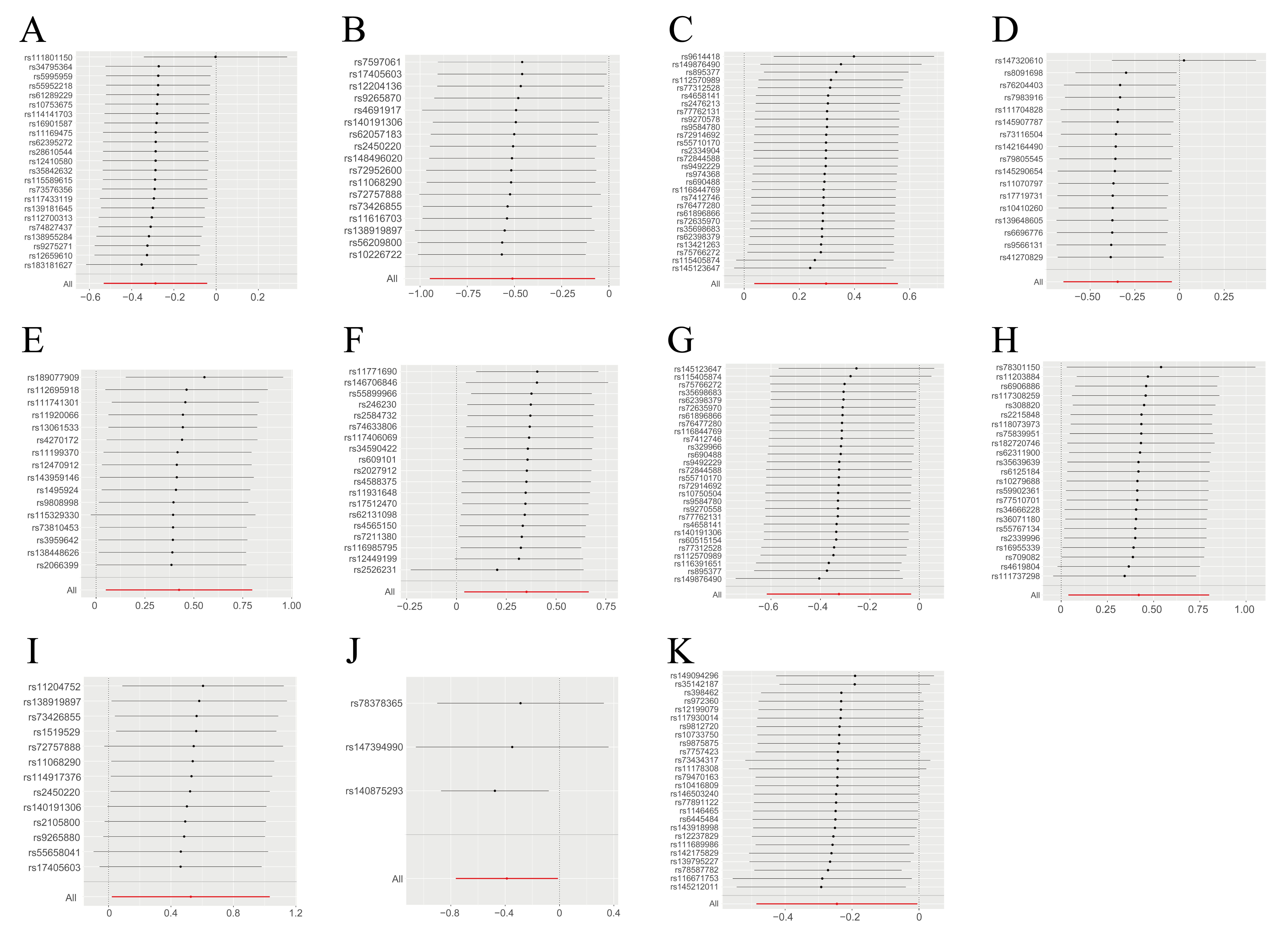


**Supplementary Fig. 4** Leave-one-out sensitivity analysis of potential causality of immune cells on CHS. **(A)** HLA DR++ monocyte AC on CHS, **(B)** IgD+ %B cell on CHS, **(C)** Naive-mature B cell %B cell on CHS, **(D)** CD45 on Mo MDSC on CHS, **(E)** CD20 on IgD+ CD38- naïve on CHS, **(F)** HLA DR on HLA DR+ T cell on CHS, **(G)** Memory B cell %B cell on CHS, **(H)** CD20- CD38- %lymphocyte on CHS, **(I)** Sw mem %B cell on CHS, **(J)** CD11b on Gr MDSC on CHS, **(K)** CD80 on CD62L+ myeloid DC on CHS.


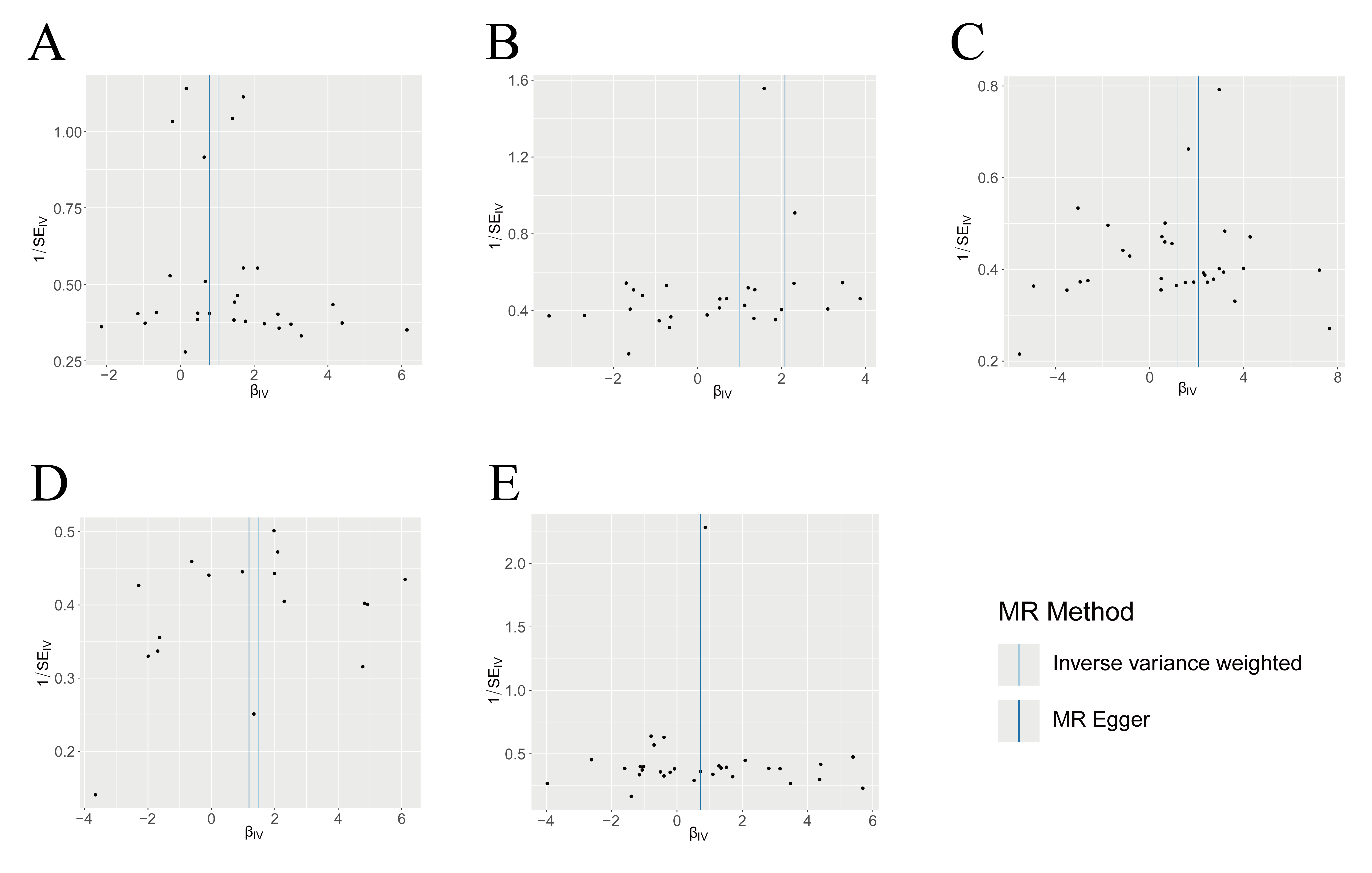
**Supplementary Fig. 5** Funnel plot of potential causality of inflammatory protein on CHS. **(A)** Monocyte chemoattractant protein-1 levels on CHS, **(B)** Leukemia inhibitory factor receptor levels on CHS, **(C)** Neurotrophin-3 levels on CHS, **(D)** Interleukin-24 levels on CHS, **(E)** C-C motif chemokine 23 levels on CHS.


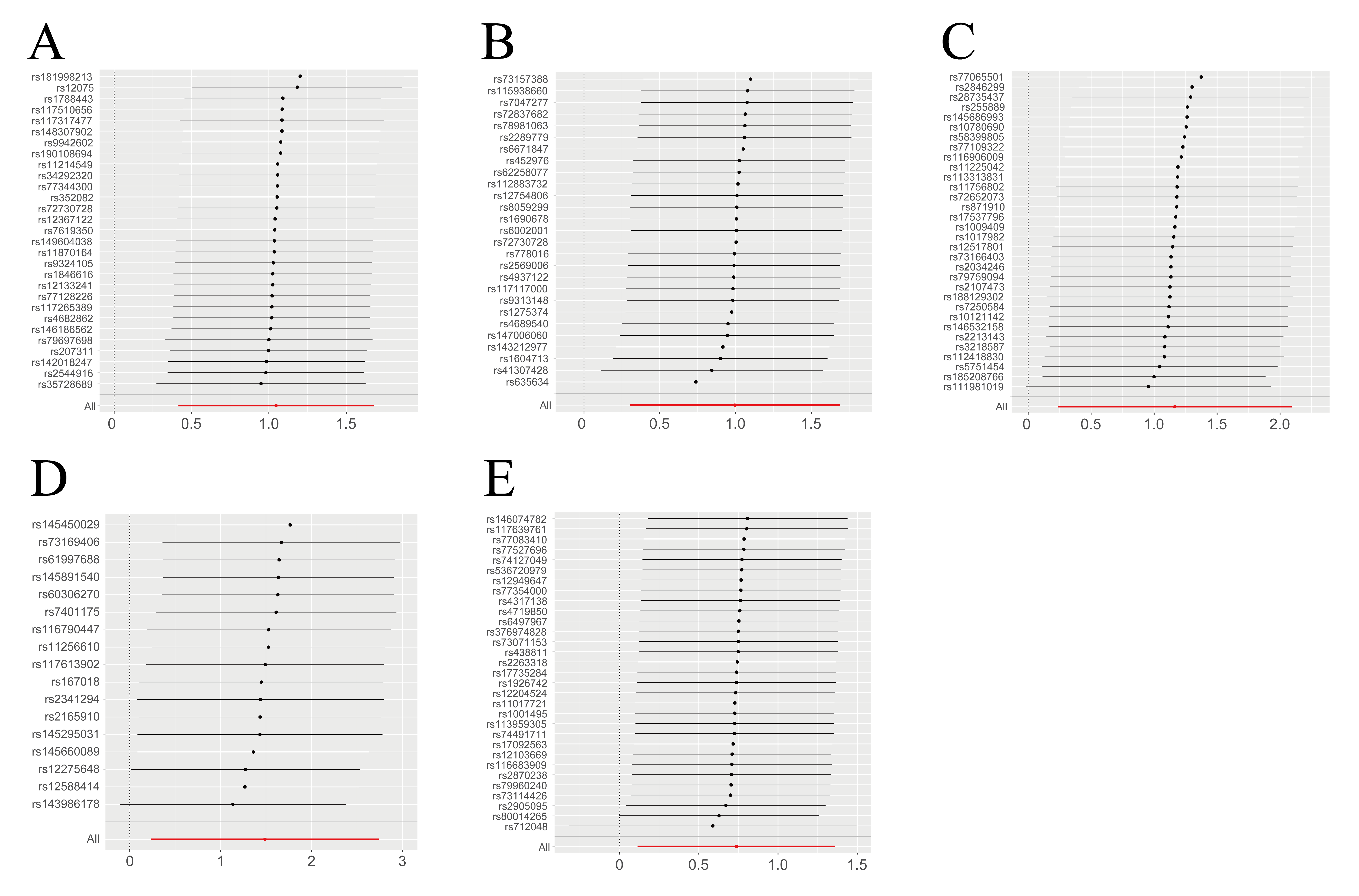


**Supplementary Fig. 6** Leave-one-out sensitivity analysis of potential causality of inflammatory protein on CHS. **(A)** Monocyte chemoattractant protein-1 levels on CHS, **(B)** Leukemia inhibitory factor receptor levels on CHS, **(C)** Neurotrophin-3 levels on CHS, **(D)** Interleukin-24 levels on CHS, **(E)** C-C motif chemokine 23 levels on CHS.
