# Supplementary material for: Exploring the causal relationship between immune factors and chondrosarcoma: a Mendelian randomization study
Source: Discov Oncol. 2025 May 18;16:801. doi: 10.1007/s12672-025-02654-5 (PMC12086138; doi:10.1007/s12672-025-02654-5)
Supplement: Supplementary file 2 — Table 1. STROBE-MR checklist of recommended items to address in reports of Mendelian randomization studies [file 12672_2025_2654_MOESM2_ESM.docx]

**Supplementary Table 1.** STROBE-MR checklist of recommended items to address in reports of Mendelian randomization studies

| **Item No.** | **Section** | **Checklist item** | **Page No.** | **Complete/location** | |
| --- | --- | --- | --- | --- | --- |
| 1 | **TITLE and ABSTRACT** | Indicate Mendelian randomization (MR) as the study’s design in the title and/or the abstract if that is a main purpose of the study | 1-2 | Complete | |
|  | **INTRODUCTION** |  |  |  | |
| 2 | **Background** | Explain the scientific background and rationale for the reported study. What is the exposure? Is a potential causal relationship between exposure and outcome plausible? Justify why MR is a helpful method to address the study question | 3-5 | The background of the study and the rationale for causality are explained in the second paragraph of the Introduction, and the concepts and principles of Mendelian randomization are described in the third paragraph of the Introduction. | |
| 3 | **Objectives** | State specific objectives clearly, including pre-specified causal hypotheses (if any). State that MR is a method that, under specific assumptions, intends to estimate causal effects | 5 | The causal question has been stated in the third paragraph of the Introduction. | |
|  | **METHODS** |  |  |  | |
| 4 | **Study design and data sources** | Present key elements of the study design early in the article. Consider including a table listing sources of data for all phases of the study. For each data source contributing to the analysis, describe the following: | 5-7 | The study design is described in the Materials and Methods section “Study design”. All data sources for the study are described in the Materials and Methods section “Data source”. | |
|  | a) | Setting: Describe the study design and the underlying population, if possible. Describe the setting, locations, and relevant dates, including periods of recruitment, exposure, follow-up, and data collection, when available. | 6-7 | The original study with GWAS data on exposure and outcomes is described in the Materials and Methods section “Data source”. | |
|  | b) | Participants: Give the eligibility criteria, and the sources and methods of selection of participants. Report the sample size, and whether any power or sample size calculations were carried out prior to the main analysis | 6-7 | All necessary information about the GWAS studies used in this study has been described in the Materials and Method section “Data source”. | |
|  | c) | Describe measurement, quality control and selection of genetic variants | 7 | Strategies for selection and quality control of genetic variants as instrumental variables are described in the Materials and Methods section “Selection of instrumental variables”. | |
|  | d) | For each exposure, outcome, and other relevant variables, describe methods of assessment and diagnostic criteria for diseases | 2-3 | A relevant description is given in the first paragraph of the Introduction. | |
|  | e) | Provide details of ethics committee approval and participant informed consent, if relevant | 5-6 | Ethical issues are explained in the Materials and Methods section “study design”. | |
| 5 | **Assumptions** | Explicitly state the three core IV assumptions for the main analysis (relevance, independence and exclusion restriction) as well assumptions for any additional or sensitivity analysis | 5-8 | The content of the three hypotheses is described in the Materials and Methods section “Study design”. The Materials and Methods section describes in detail the process of instrumental variable extraction and validation of correlation assumption through *F*-statistics, and the exclusion restriction assumption was detected by sensitivity analysis methods such as MR-Egger regression, weighted median, and weighted mode analysis. | |
| 6 | **Statistical methods: main analysis** | Describe statistical methods and statistics used  a) Describe how quantitative variables were handled in the analyses (i.e., scale, units, model)  b) Describe how genetic variants were handled in the analyses and, if applicable, how their weights were selected  c) Describe the MR estimator (e.g. two-stage least squares, Wald ratio) and related statistics. Detail the included covariates and, in case of two-sample MR, whether the same covariate set was used for adjustment in the two samples  d) Explain how missing data were addressed  e) If applicable, indicate how multiple testing was addressed | 7-8 | | All necessary information about the statistical methods and statistics in this study has been described in the Materials and Methods section “Mendelian randomization analysis and sensitivity analysis”. |
|  | |  |  |  |  |
| 7 | **Assessment of assumptions** | Describe any methods or prior knowledge used to assess the assumptions or justify their validity | 5-8 | All necessary information is described in the Materials and Methods section. | |
| 8 | **Sensitivity analyses and additional analyses** | Describe any sensitivity analyses or additional analyses performed (e.g. comparison of effect estimates from different approaches, independent replication, bias analytic techniques, validation of instruments, simulations) | 7-8 | Sensitivity analyses and additional analyses are presented in the Materials and Methods section “Mendelian randomization analysis and sensitivity analysis”. | |
| 9 | **Software and pre-registration** |  |  |  | |
|  | a) | Name statistical software and package(s), including version and settings used | 5-8 | All statistical software and settings used are described in the Materials and Methods section. | |
|  | b) | State whether the study protocol and details were pre-registered (as well as when and where) | 5-6 | The analysis plan was described in the " Study design” of the Materials and Methods section. | |
|  | **RESULTS** |  |  |  | |
| 10 | **Descriptive data** |  |  |  | |
|  | a) | Report the numbers of individuals at each stage of included studies and reasons for exclusion. Consider use of a flow diagram | / | Details of the inclusion and exclusion of instrumental variables in the study are described in Table S3 and Table S5. | |
|  | b) | Report summary statistics for phenotypic exposure(s), outcome(s), and other relevant variables (e.g. means, SDs, proportions) | 5-8 | We described the detailed information of the summary statistics for our analysis in the Materials and Methods section. | |
|  | c) | If the data sources include meta-analyses of previous studies, provide the assessments of heterogeneity across these studies | / | The results of the heterogeneity assessment are shown in Table S2 and Table S4. | |
|  | d) | For two-sample MR:  i.  Provide justification of the similarity of the genetic variant-exposure associations between the exposure and outcome samples  ii.  Provide information on the number of individuals who overlap between the exposure and outcome studies | 5-8 | We provide this information in the section of Materials and Methods section. | |
| 11 | **Main results** |  |  |  | |
|  | a) | Report the associations between genetic variant and exposure, and between genetic variant and outcome, preferably on an interpretable scale | / | The associations between the genetic variant and exposure, and between the genetic variant and outcome have been reported in Table S3 and Table S5. | |
|  | b) | Report MR estimates of the relationship between exposure and outcome, and the measures of uncertainty from the MR analysis, on an interpretable scale, such as odds ratio or relative risk per SD difference | / | The causal effect estimates between exposures and outcomes are listed in Figures 2-6, Table S2, and Table S4. | |
|  | c) | If relevant, consider translating estimates of relative risk into absolute risk for a meaningful time period | / | Our results were presented in terms of odds ratio and confidence intervals throughout the Results section for quantitative outcomes. | |
|  | d) | Consider plots to visualize results (e.g. forest plot, scatterplot of associations between genetic variants and outcome versus between genetic variants and exposure) | / | Information on the associations between genetic variation and exposure is presented in Table S3 and Table S5, and the scatter plots of the associations between genetic associations and outcomes are presented in Figure 3, Figure 4, and Figure 6. | |
| 12 | **Assessment of assumptions** |  |  |  | |
|  | a) | Report the assessment of the validity of the assumptions | 9-14 | We assess the validity using sensitivity analyses, MR Egger regression, weighted median and weighted mode approach. Results were presented in the Results section. | |
|  | b) | Report any additional statistics (e.g., assessments of heterogeneity across genetic variants, such as *I^2^*, Q statistic or E-value) | / | The results of Cochran's Q heterogeneity test are in Table S2 and Table S4. | |
| 13 | **Sensitivity analyses and additional analyses** |  |  |  | |
|  | a) | Report any sensitivity analyses to assess the robustness of the main results to violations of the assumptions | 9-14 | We reported the use of additional MR methods and sensitivity analyses as additional approaches to validate the main results in the Results section. | |
|  | b) | Report results from other sensitivity analyses or additional analyses (e.g., replication study with different dataset, analyses of subgroups, validation of instrument(s), simulations, etc.) | / | The validation of instruments was reported in Table S3 and Table S5. | |
|  | c) | Report any assessment of direction of causal relationship (e.g., bidirectional MR) | / | Due to insufficient instrumental variables, we only did one-way MR and detected the direction of causal inference by MR Steiger test. | |
|  | d) | When relevant, report and compare with estimates from non-MR analyses | NA | Irrelevant | |
|  | e) | Consider additional plots to visualize results (e.g., leave-one-out analyses) | / | The results of the leave-one-out analyses are shown in Figure S3, Figure S4, and Figure S6. | |
|  | **DISCUSSION** |  |  |  | |
| 14 | **Key results** | Summarize key results with reference to study objectives | 14 | Discussion paragraph 1 | |
| 15 | **Limitations** | Discuss limitations of the study, taking into account the validity of the IV assumptions, other sources of potential bias, and imprecision. Discuss both direction and magnitude of any potential bias and any efforts to address them | 19 | The final paragraph of the Discussion provides a discussion of the limitations of the study. | |
| 16 | **Interpretation** |  |  |  | |
|  | a) | Meaning: Give a cautious overall interpretation of results in the context of their limitations and in comparison with other studies | 15-19 | Discussion paragraphs 2-3 | |
|  | b) | Mechanism: Discuss underlying biological mechanisms that could drive a potential causal relationship between the investigated exposure and the outcome, and whether the gene-environment equivalence assumption is reasonable. Use causal language carefully, clarifying that IV estimates may provide causal effects only under certain assumptions | 15-19 | Discussion paragraphs 2-3 | |
|  | c) | Clinical relevance: Discuss whether the results have clinical or public policy relevance, and to what extent they inform effect sizes of possible interventions | 19 | We describe the clinical relevance in the Conclusion section. | |
| 17 | **Generalizability** | Discuss the generalizability of the study results (a) to other populations, (b) across other exposure periods/timings, and (c) across other levels of exposure | 19 | We have discussed the potential caveats in terms of the generalizability of our findings in the final paragraph of the Discussion. | |
|  | **OTHER INFORMATION** |  |  |  | |
| 18 | **Funding** | Describe sources of funding and the role of funders in the present study and, if applicable, sources of funding for the databases and original study or studies on which the present study is based | 20 | We have reported all sources of funding in the “Acknowledgements” section. | |
| 19 | **Data and data sharing** | Provide the data used to perform all analyses or report where and how the data can be accessed, and reference these sources in the article. Provide the statistical code needed to reproduce the results in the article, or report whether the code is publicly accessible and if so, where | 20 | We have provided the link/approach to access genetic data used in this study in the "Data availability" section. | |
| 20 | **Conflicts of Interest** | All authors should declare all potential conflicts of interest | 20 | We have declared conflicts of interest in the “Competing interests” section. | |
